# AI-Driven Drug Discovery: A Comprehensive Review

**DOI:** 10.1021/acsomega.5c00549

**Published:** 2025-06-06

**Authors:** Fábio J. N. Ferreira, Agnaldo S. Carneiro

**Affiliations:** 37871 Universidade Federal do Pará, R. Augusto Corrêa, 01 - Guamá, Belém, Pará 66075-110, Brazil

## Abstract

Artificial intelligence (AI) and machine learning (ML) offer transformative potential to address the persistent challenges of traditional drug discovery, characterized by high costs, lengthy timelines, and low success rates. This comprehensive review critically analyzes recent advancements (2019–2024) in AI/ML methodologies across the entire drug discovery pipeline, from target identification to clinical development. We examine diverse AI techniques, including deep learning, graph neural networks, and transformers, focusing on their application in key areas such as target identification, lead discovery, hit optimization, and preclinical safety assessment. Our in-depth comparative analysis highlights the advantages, limitations, and practical challenges associated with different AI approaches, emphasizing critical factors for successful implementation such as data quality, model validation, and ethical considerations. The review synthesizes current applications, identifies persistent gaps—particularly in data accessibility, interpretability, and clinical translation—and proposes future directions to unlock the full potential of AI in creating safer, more effective, and accessible medicines. By emphasizing transparent methodologies, robust validation, and ethical frameworks, this review aims to guide the responsible and impactful integration of AI into pharmaceutical research and development.

## Graphical abstract

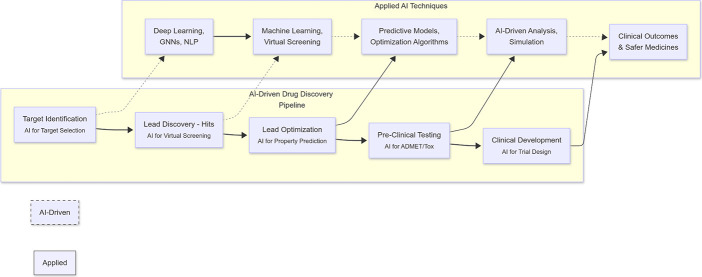

## 1 Introduction

The traditional drug discovery process is complex, costly, and time-consuming, often spanning over a decade and exceeding 2 billion.^1–3^ This is mainly due to the sequential nature of its stages, involving target identification, hit discovery, lead optimization, preclinical testing, and lengthy clinical trials that all require vast resources and validation. Critically, the process suffers from a low success rate, as only approximately 10% of drugs that enter clinical trials ultimately achieve regulatory approval, often exacerbated by high attrition rates^2,4^ from safety concerns and a lack of efficacy. Further, high-throughput screening (HTS), a common method, yields only a 2.5% hit rate, which further lengthens timelines, increases cost, and wastes resources.^5^

These challenges demand more efficient methods, where artificial intelligence (AI) and machine learning (ML) offer a promising path toward increased efficiency and success rates in drug development, providing the pharmaceutical industry with a solution with AI/ML implementation to correct limitations while also opening up novel opportunities using new model implementations based on AI parameters.^6^

### 1.1 Objectives of the Review

This literature review critically analyzes AI/ML applications in drug discovery by (1) summarizing current AI/ML utilization and highlighting methodologies that impact current paradigms; (2) identifying key AI/ML techniques and their implementation parameters across different drug development steps; (3) assessing the impact of those methods, addressing their limitations/challenges, and showing new paths for optimizations; and (4) evaluating new trends as large language model implementations in the context of ethics, bias, data access, and regulatory parameters. While numerous reviews have explored AI applications in drug discovery, this comprehensive review distinguishes itself by its in-depth comparative analysis of AI methodologies across the entire drug discovery pipeline, from target identification to clinical development, with a particular emphasis on practical challenges and future directions and its critical evaluation of the integration of diverse data types and model architectures, providing a nuanced perspective on their strengths and limitations.

### 1.2 Scope and Boundaries

This review comprehensively analyzes recent advancements (2019–2024) in artificial intelligence and machine learning (AI/ML) applied to drug discovery. The scope emphasizes cloud-based implementations and modern model architectures particularly relevant to pharmaceutical industry applications. Examining English-language publications globally, encompassing research from diverse international teams, this study primarily targets AI implementations for small molecule drug discovery within key areas: Target Identification, Hit/Lead Discovery & Optimization, and Preclinical Analysis & Safety. Emphasis is placed on *in silico* and computational methods employing AI for design and analysis of chemical/biological properties and target selection. Studies focused *solely* on formulation, automation, or robotics/high throughput are excluded unless they incorporate *direct* AI implementations. For enhanced robustness and scientific rigor, specific implementations using only single-patient data sets or non-peer-reviewed materials (preprints) are generally excluded; however, exceptionally novel or significantly relevant preprints *may* be considered depending on their specific novelty and importance.

### 1.3 Key Concepts and Terminology

This review centers on the application of AI and ML within drug discovery. AI, broadly defined, encompasses the simulation of human intelligence through computer-based models. In the context of drug discovery, AI utilizes ML, a subfield focused on enabling systems to learn from data, predict outcomes, and generate outputs without explicit programming. Deep learning (DL), a sophisticated subset of ML, employs artificial neural networks with multiple layers to analyze complex, unstructured data and solve intricate problems. These AI/ML tools are applied across critical stages of drug development, including: Target Identification, where AI accelerates the discovery of relevant biological targets in disease through advanced computational analysis of biological data sets; Lead Optimization, leveraging AI/ML to refine drug candidates by improving bioactivity, chemical properties, and structure; ADME-Tox Studies, utilizing AI-based evaluations to predict drug absorption, distribution, metabolism, excretion, and potential toxicity; High Throughput Screening (HTS), where AI enhances assay systems to efficiently identify promising compounds from large libraries; and Drug Repurposing, employing AI to find new therapeutic uses for existing compounds initially developed for different indications.

Understanding pharmacological principles is crucial in this context. Bioactivity refers to a drug or ligand’s measurable impact on biological systems at the cellular or organismal level. Ligands, encompassing drugs, molecules, or specific elements, interact with biological components, primarily proteins. Drug-likeness models guide compound selection by assessing if molecules possess properties aligning with favorable clinical and pharmacological characteristics, increasing their chances of successful drug development. Pharmacokinetic Profiles, often summarized by ADME parameters, characterize how drugs behave within biological systems. Finally, Clinical Outcomes, measured by direct feedback in patient settings (e.g., health, quality of life, survival), quantify the ultimate effectiveness of a drug in real-world scenarios.

### 1.4 Review Organization

This review provides a structured analysis of AI implementation in pharmaceuticals. It begins by introducing the context, then outlining literature search strategies and highlighting key theoretical aspects. Subsequently, it evaluates diverse AI applications across distinct phases of drug development, emphasizing model validation parameters and presenting findings related to each phase. Ultimately, based on synthesized data, this review delivers a focused overview, pinpointing existing gaps, projecting future research directions by analyzing current trends, and concluding with a reflection on the scientific, ethical, and practical implications of AI implementations within complex pharmaceutical systems.

## 2 Methods

This section outlines the rigorous and repeatable methodology used to search for and select relevant peer-reviewed publications, ensuring a reliable data set for this literature review.

### 2.1 Search Strategy

A comprehensive systematic search was performed across PubMed, Scopus, Web of Science, and Google Scholar to identify publications concerning AI and ML in small molecule drug discovery. The search strings were designed based on a PICO framework (P - Drug Discovery, I - AI/ML, and O - Results/implementation outputs), combining relevant keywords; see Table 1 for the complete keyword list. These strings utilized Boolean operators (AND/OR) to connect keywords for more precise selections of data sets. The database search utilized filters (year of publication and language) that focused mostly on recently published papers to represent current trends/perspectives. All selected publications were in the English language.

**Table 1:** Full Search of Keywords Implementation with Key Phrases Relating to the PICO Protocol Used during Data Extraction

Category	Keywords/Phrases
Core AI/ML Terms	“artificial intelligence”, “machine learning”, “deep learning”, “neural networks”, “AI algorithms”, “AI”, “ML”, “DL”
Drug Discovery	“drug discovery”, “drug development”, “pharmaceutical research”, “target identification”, “hit identification”, “lead optimization”, “virtual screening”, “drug repurposing”, “cheminformatics”, “ADME”, “toxicology prediction”, “clinical trials design”, “de novo drug design”, “high-throughput screening”
Drug Discovery (General)	“pharmaceutical sciences”, “medicinal chemistry”, “in silico drug design”, “in silico”
Target and Data Set Terms	“transcriptomics”, “genomics”, “proteomics”, “biological networks”, “disease targets”, “biomarkers”, “target validation”, “bioactivity”, “ligand binding”
Relevant Methodologies	“convolutional neural networks”, “graph neural networks”, “transformer neural networks”, “generative AI”
Relevant Concepts	“model evaluation”, “bias assessment”, “explainable AI”, “data accessibility”, “ethics”

The database search was performed until December 24, 2024, encompassing studies from January 1, 2019, to capture more recent advances in the field.

### 2.2 Inclusion and Exclusion Criteria

This review encompassed peer-reviewed full research articles focused on AI/ML methodologies in small molecule drug discovery as the primary focus, supplemented by select high-impact meta-analyses, reviews, and key data sets. Included studies presented AI-driven methodologies in areas such as target identification, lead optimization, ADMET/toxicity prediction, clinical studies, and drug repurposing, emphasizing implementation models with parameters validating scientific and biological relevance. Articles utilizing *in silico* or hybrid theoretical/computational methods and models, particularly those centered on data and model analyses with AI, were prioritized, while excluding studies primarily focused on robotic automation or screening lacking clear AI model descriptions or end point translation.

Conversely, opinion pieces, editorials, letters without empirical data, and abstract-only publications (without accompanying full-text publications) were generally excluded (with few limited exceptions when data was highly relevant and needed for critical discussion). However, in recognition of the rapid pace of advancements in AI/ML and the potential for timely dissemination of important findings via preprint servers, highly relevant preprint articles, particularly those from reputable sources such as arXiv, and directly pertinent to the review’s scope, were considered for inclusion on a case-by-case basis, if they offered significant and validated insights not yet available in peer-reviewed literature. This exception aimed to ensure the review captured the most cutting-edge developments in this dynamic field, without compromising overall rigor and focus on robust scientific findings. Also excluded were reports discussing general AI without focused ML implementation parameters in pharmaceuticals, and studies with limited data sets derived from case studies or small patient groups. Non-English language publications and studies focused solely on automation without direct AI integration were also omitted from this review. Duplicates were resolved using EndNote and manual review, prioritizing higher-impact publications with clearer data and methods to represent each unique model and to ensure comprehensive data synthesis.

### 2.3 Study Selection Process

#### 2.3.1 Citation Management

Citations from database search results were cataloged with EndNote (version X20). Duplicates were removed first via software and also by manually reviewing each selected article. Relevant articles were tracked during selection. New reports found outside the core search were tracked by being manually introduced to the data set to avoid overlooking or not finding any important contributions that were discovered while building up the search database for evaluation purposes.

#### 2.3.2 Article Identification and PRISMA Flow Diagram

A PRISMA 2020 diagram (see Figure 1) was generated based on the inclusion-exclusion protocol. The steps involved were: identification of all articles from previously selected database; screening, where data was assessed using titles/abstract and inclusion/exclusion criteria (as described in Sections 2.2 and 2.3) based on PICO framework; eligibility analysis to access to methodology and main results by reading the full document text; publications included for synthesis for results assessment and final analyses.

**Figure 1:**
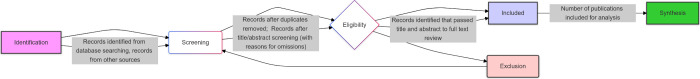
Stages followed for this literature review included an initial search data collection and duplicate removal step using specific databases, followed by a title and abstract review that was performed using predefined inclusion and exclusion parameters, as well as all methodology for full article assessment/classification for data synthesis from published reports (utilizing a PICO framework based data implementation strategy for robust data validation).

#### 2.3.3 Screening Process

A lead author performed initial screening (title, abstract). All studies that passed screening were also assessed in full for inclusion by the same researcher and verified with a coauthor, including methodology assessments to avoid overlooking or data implementation errors. All reasons and limitations were clearly noted and saved for full transparency (as stated during data access).

### 2.4 Quality Assessment/Risk of Bias

Recognizing the limitations of standardized checklists for the rapidly evolving field of AI/ML in pharmaceutical science, this review prioritized scientific transparency for quality assessment over predefined questionnaires. The evaluation focused on methodological rigor and translatability, specifically targeting studies with (a) clear parameter implementation; (b) scientifically sound, translatable models robustly assessed using open data sets/code; and (c) explicit discussions of biases and limitations. Key quality parameters included: (a) translatability of methods and data; (b) clarity of methodological steps and parameter implementation details (data set usage, code/model accessibility); and (c) thorough analysis of inherent limitations/biases in study design, implementation, and evaluation, favoring reproducible results with high testability. This emphasis on core scientific components throughout the evaluation process aimed to mitigate bias and ensure an accurate assessment of each publication.

## 3 Theoretical Framework

This section establishes the theoretical foundation and historical background for AI in drug discovery, emphasizing the evolution of modern algorithms and models, their applicability, key researchers/institutions influencing the field, and related ethical/regulatory implications, setting the stage for a detailed data synthesis that follows in subsequent sections.

### 3.1 Historical Evolution of AI in Drug Discovery

The history of Artificial Intelligence (AI) in drug discovery begins with early computational methods in the 1960s and 1970s where computer-aided drug design (CADD) emerged to improve efficiency. Initial approaches involved *quantitative structure–activity relationship* (QSAR) models, using statistics to correlate chemical structure and biological activity, laying the foundation for more advanced techniques and showing that algorithms could be a tool for drug development and design.^7^

In the 1980s and 1990s, alongside increasing computational power enabling molecular docking and virtual screening, ML methods began to emerge as valuable tools in drug discovery, particularly in the realm of QSARs. Early QSAR approaches, evolving from methods like Hansch analysis^8^ that used statistical linear models, started to incorporate machine learning algorithms such as Random Forests^9^ and Support Vector Machines (SVMs)^10^ to model complex relationships between molecular structure and biological activity using industrial data sets. These methods marked a shift from purely statistical correlations toward more sophisticated, data-driven approaches for drug design and prediction.^11^

With ML, algorithms were implemented to process larger data sets, further improving AI analysis power to select potential candidates for drug testing. Finally in the early 2000s, deep learning (DL) algorithms enabled more complex data models (with complex data structures), which led to an enhanced ability to analyze complex relationships between chemical/biological information in a data-driven approach based on high throughput/multi parameter analyses while modeling and assessing drug–target interactions and improving screening methods based on AI parameters.^12^ These new AI-based parameters allow more detailed analysis of data for complex pharmaceutical questions in preclinical/clinical phases. Current AI approaches highlight the use of pattern analysis over vast amounts of diverse data set information and are applicable through all steps of the drug discovery pipeline. AI is also considered a powerful instrument to reduce bottlenecks in preclinical and clinical phases.^13^ The recent advances in artificial intelligence are catalyzing a paradigm shift in the pharmaceutical industry. AI-based methods are now routinely deployed in laboratories worldwide to automate and optimize experimental workflows, driving a scientific revolution in drug discovery. By integrating large, multidisciplinary data sets—including molecular structures, disease-progression metrics, treatment modalities, and patient-outcome records—these approaches enable the development of novel therapeutics beyond traditional compound-centric design strategies.

### 3.2 Core AI/ML Paradigms and Their Applicability

The range of AI/ML tools utilized in drug discovery encompasses a spectrum of methodologies, from classic to cutting-edge, each with distinct advantages, limitations, and varying use in distinct drug development stages. The landscape of AI/ML tools for drug discovery is increasingly dominated by Graph Neural Networks (GNNs) and Transformer architectures. These modern methodologies have demonstrated remarkable success in learning complex molecular representations and achieving state-of-the-art performance on a variety of crucial drug discovery tasks. While classical ML methods retain value for certain applications, GNNs and Transformers represent the cutting edge in capturing intricate structural and relational information essential for advanced drug design and prediction. While initial graph transformers relied heavily on specialized encodings, newer approaches are exploring alternative paradigms. For instance, Edge-Set Attention (ESA) treats graphs purely as sets of edges and leverages interleaved masked and standard attention mechanisms, demonstrating strong performance without complex positional or structural encodings.^14^

#### 3.2.1 Graph Neural Networks (GNNs) in Molecular Modeling

GNNs have emerged as particularly powerful tools in molecular modeling due to their ability to directly learn from the graph-based structure of molecules, capturing complex relationships between atoms and bonds. To illustrate the architecture of a GravNet^15^ block within a GNN, see Figure 2.

**Figure 2:**
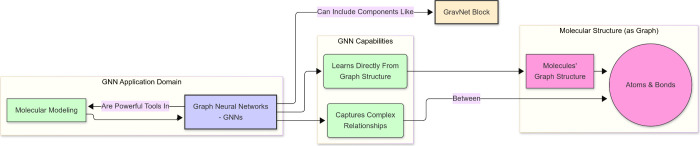
Schematic overview of the application of Graph Neural Networks (GNNs) in molecular modeling. The figure highlights: (i) the application domain; (ii) the key capabilities of GNNs, namely, direct learning from the graph structure and capturing complex relationships; (iii) the representation of molecular structure as a graph comprising atoms and bonds; and (iv) the integration of specific architectural blocks, such as GravNet, within the GNN framework.

Architectures like Graph Convolutional Networks (GCNs)^16^ and Graph Attention Networks (GATs)^17^ and newer variants like Principal Neighborhood Aggregation (PNA)^18^ have shown exceptional performance on key molecular data sets. For instance, state-of-the-art GNN models have achieved impressive results on benchmarks like MoleculeNet,^19^ demonstrating strong performance on data sets like ESOL^20,21^ (solubility prediction) and FreeSolv^22^ (hydration free energy prediction). Furthermore, models benchmarked on data sets like DOCKSTRING targets^23^ (e.g., PGR, F2, KIT targets) highlight the capacity of GNNs to capture complex protein–ligand interactions relevant to drug discovery.

Recent benchmarking studies further emphasize the crucial role of architectural choices in GNN performance. For example, Grötschla et al.^24^ conducted a comprehensive analysis of positional encodings across diverse GNN architectures, including Graph Transformers, revealing valuable insights into the impact of positional encodings on model performance. Their findings, derived from extensive benchmarking across data sets like GNN BENCHMARKING^25^ and LRGB,^26^ highlight the importance of carefully selecting positional encodings to optimize GNN performance and demonstrate that different positional encodings can lead to significant variations in results depending on the data set and task. Such studies underscore the ongoing efforts to optimize GNN architectures and guide practical model selection in drug discovery and beyond.

#### 3.2.2 Transformer Architectures in Molecular Modeling

Transformer architectures, initially revolutionizing Natural Language Processing, are now making significant inroads into molecular machine learning. Their ability to model long-range dependencies and complex contextual relationships through attention mechanisms is highly valuable for capturing nuanced molecular properties and interactions. Models like Mol-BERT^27^ and adaptations of Transformers specifically tailored for graph data, such as Graph Transformers,^28^ have shown promise on tasks like PCQM4Mv2 (quantum chemistry property prediction) and in predicting peptide properties on the LRGB-Peptides data set.^26^ Ongoing research explores the optimal integration of Transformers with graph-based methods to leverage the strengths of both architectures for molecular representation learning.

Classical ML techniques, like Decision Trees^29^ and the more robust Random Forest,^30^ offer accessible data visualization. Support Vector Machines (SVMs) excel at strong data classifications, while K-Nearest Neighbors (KNN) provides a simpler starting point for basic classification problems.^31,32^ Deep learning (DL), representing models beyond GNNs and Transformers and utilizing complex artificial neural networks, enables advanced data analysis for feature identification, structural and molecular property prediction, and parameter optimization, particularly with large data sets, facilitating the creation of potent drug–target models.^33^ Reinforcement learning (RL) is employed in systems requiring sequential data evaluation and feedback-driven outcome optimization.^34^ All methods present specific capabilities in data implementation depending on the design goal or data evaluation criteria/types used by each different parameter selection during study implementation.

The choice of AI/ML method is intrinsically linked to the design goal, data characteristics, and evaluation criteria defined by parameter selection in each study. Model validation is crucial for study validity, as data quality directly impacts bias, and a limited understanding of DL may result in interpretability issues that need robust methodology to make real data validations during complex analyses.^35,36^ The architecture of ML models, encompassing the methods discussed above (GNNs, CNNs, Transformers, etc.) is paramount for effective model evaluation. Methodological choices, therefore, strongly depend on data set structure, parameters, study design, available computational resources, and institutional expertise.

Selecting appropriate AI methods in drug discovery hinges on aligning the chosen approach with the specific evaluation stage and ensuring clinically relevant and interpretable results.^13,37^ Classical ML remains effective in early stage target selection. Deep learning, including GNNs and Transformers, facilitates complex modeling and target/drug prioritization. Network implementations are valuable for analyzing drug interaction data and analyses, particularly from complex, highly interconnected data sets mirroring biological systems. Method selection is further influenced by the availability of resources, training in AI programming and mathematics, and the specific demands of experimental approaches.

### 3.3 Ethical and Regulatory Considerations

The growing integration of AI and ML in drug development necessitates proactive management of ethical and regulatory challenges to ensure safe applications. Data bias represents a primary concern, potentially skewing model predictions, exacerbating demographic inequities, and demanding enhanced data parameters and rigorous testing protocols. Robust model validation is also critical for predictability and real-world applicability, requiring well-established models and effective bias control. Transparency in data sets and implementation parameters is equally essential to overcome “black box” limitations, foster trust in AI-derived results, and guarantee patient benefit. In response, regulatory bodies like the FDA and EMA are actively developing AI safety parameters and promoting diverse population validation, informed by detailed regulatory guidelines for robust, ethical AI technologies. Future progress relies on collaborative engagement among scientists, industry, ethicists, and policymakers to effectively address potential biases and risks within AI-driven drug development, thereby ensuring responsible innovation in drug research and testing.^38–40^

## 4 Review of Findings and Discussions

This section synthesizes the review’s findings based on different AI-based methodology applications within core steps of pharmaceutical research. In all subsections, model limitations, biases, and validation parameters will also be evaluated for their impact in all phases. We will discuss each specific type of AI implementation based on information extracted from previous reports, while evaluating the opportunities and future perspectives that emerge from those analyses, such as an integrated and core evaluation for model effectiveness.

### 4.1 Theme 1: Methodologies Used for Drug–Target Identification

AI-based tools have enabled faster and more robust ways for researchers to identify drug targets, previously overlooked using classical methods in scientific knowledge and biological/chemical data set interpretations. NLP (Natural Language Processing), AI for Omics Data Analysis, Molecular Similarity, and Network Pharmacology approaches are assessed for benefits, as well as areas needing further exploration in their respective methods of data interpretations based on what has been shown in recent reports.

#### 4.1.1 AI Methodologies for Target Identification in Drug Discovery

- **NLP:** Using vast textual data sets from published scientific and pharmaceutical documentation is key to implementing powerful AI-driven methodology and frameworks using Named Entity Recognition, Relationship Extraction, and model based statistical evaluation. The process starts by a careful extraction of raw unstructured data (from scientific texts), that can then be processed to extract specific molecular/biological interactions using named entity recognizers for the identification of specific drugs, genes, and proteins such as a base. Implementation also focuses on network development based on these data to perform predictive analytics and novel drug discovery through pathway mapping and novel target identifications.^41–44^ Although data are accessible through public databases, current NLP AI methods lack capabilities for proper evaluations on biases introduced by language representation or text source variability (which often only include scientific results with a more specific context than clinical practice), and context recognition remains such as one of the key limiting steps to translate AI discoveries into real life solutions that are based on more than only text-driven implementations.
- **AI in Omics Data Analysis:** AI and ML models provide robust approaches for processing complex data generated in genomics, transcriptomics, and proteomics. Utilizing both supervised and unsupervised learning techniques, these models effectively handle large, high-dimensional data sets that traditional methods struggle to analyze. During model implementation, data sets typically undergo preprocessing steps to eliminate biases and redundancies while emphasizing statistically significant features. Unsupervised methods focus on identifying patterns and groupings within the data, uncovering associations with diseases and facilitating predictive evaluations and biomarker identification.^45–47^ Further validation involves cross-linking with known pathways to add to their scientific validation value. Limitations for data sets come from variability in source parameters and from a lack of clear/standardized methodologies. Data sets with complex and noisy values are common in omics areas of science, and AI interpretation needs strong experimental parameters to translate model parameters to biological/clinical settings to fully realize its high data mining implementation (if done correctly).
- **Molecular Similarity:** AI tools leverage chemical data using methods such as Tanimoto Coefficients or cosine similarity coupled with the implementation of descriptors or molecular fingerprints to identify relevant components relating molecules to previously known drug ligands based on structure similarity and ligand–receptor complex interactions.^48^ Further models, built upon deep learning approaches (like Graph Neural Networks), use more complex features to model ligand interactions or can test new chemical regions for specific properties, thus creating a system that implements models with high precision but also a potential bias of limiting selection based only on well explored molecular regions. Also, model implementation needs high quality information about data set structure (which is publicly accessible in databases such as PUBChem, ZINC, and ChEMBL) that becomes a bottleneck when specific chemical compounds and/or novel structures are being screened using new data sets outside available open data sets, since model reliability will be tested more extensively against familiar chemical space.^49,50^

- **Network Pharmacology**: Using graph-theoretical concepts alongside AI allows for more precise target discovery that uses not just single aspects (or just structure) but system-based evaluations with interrelations using multiple types of data outputs. Key aspects use network maps of protein–protein interaction, pathways (KEGG/GO), and genomic data sets that are combined with a Graph Neural Networks (GNNs) for better selection of strategic targets that influence large portions of cellular pathways based on a complete topological map, but this has the potential for biases if specific mechanisms or molecules are underrepresented in the known public databases, or with limitations in evaluations of parameters or data sets not used by original implemented models.^51^

#### 4.1.2 Model Architectures Applied in Target Identification

For target identification, diverse model architectures are employed depending on the data source and desired output. For text processing of literature and biological databases, Transformer-based architectures, particularly adaptations of BERT, are increasingly prevalent. These models leverage self-attention mechanisms to effectively capture long-range dependencies within textual data, crucial for understanding complex biological contexts.^27^ For instance, Mol-BERT, a BERT architecture pretrained on 4 million unlabeled drug molecules, creates contextualized embeddings that capture latent chemical rules beneficial for target identification and downstream tasks. Parameter considerations for Transformers often include the number of layers (e.g., a 12-layer architecture in Mol-BERT), attention heads (e.g., multihead attention with 8–12 heads), and embedding dimensions (e.g., 768-dimensional embeddings in Mol-BERT).^27,52^

For molecular and network data sets relevant to target identification, GNNs, especially Graph Convolutional Networks (GCNs) and Graph Attention Networks (GATs), have shown remarkable efficacy.^52–54^ Random Walk Guided GNN (RWGNN), for example, integrates random walk profiles with graph convolutions to predict distant drug–target interactions, achieving an AUC of 0.957 for DTIs ≥ 3 hops away, outperforming standard GCNs.^55^ Another architecture, DTI-HETA, constructs heterogeneous graphs integrating drug, target, and known DTI data and uses GAT layers to learn node embeddings, achieving state-of-the-art performance in DTI prediction.^53^ Multilayer perceptron (MLP) models and classical algorithms also remain relevant, especially for simpler data sets or initial exploratory analyses in target prioritization.

Recent advancements continue to refine AI-driven DTI prediction with sophisticated model architectures. For example, Wei et al.^56^ recently proposed EADTN, an efficient deep model ensemble framework leveraging a novel feature adaptation technique and clustering-enhanced fine-tuning to improve the accuracy and efficiency of DTI prediction. This work underscores the ongoing innovation in model design within the field.

#### 4.1.3 Model Evaluation Criteria for Target Identification

Evaluations of model performance use accuracy values (sensitivity/specificity data, AUC and F1 scores for better handling bias data set implementation). Model design parameters (including pre/post processing steps for data sets, hyperparameter and design evaluations, and algorithm characteristics) are tested using k-fold validation (for stability and generalizability assessment across diverse/new data implementations) while reporting performance across diverse, validation, and original data sets, in original reports to test all performance-based validation results. Data sets with full translatable access also indicate better methodologies with robust models that provide higher scientific translatability/implementation.^57^

#### 4.1.4 Advantages, Disadvantages, Limitations and Opportunities

The previous parameters and data information provide the following insights on key limitations, advantages, and opportunity areas: NLP offers high speed, large data handling capacity for target identification, but may require more detailed interpretation protocols based on context/data variations, limiting its implementation when the data are complex or are far from a previously documented scientific study (with the use of open language or more colloquial settings). *Omics* methods produce excellent high throughput/multi parametric evaluations of data sets allowing complex drug–target pathway interpretations; however, it faces problems due to biases from source, methodology, and type of data collected by laboratories (that lack direct standardization across institutions), making clinical implementations highly variable when transferring data from one source to another. Thus, new implementation models must consider clinical aspects to produce validated therapeutic models. In contrast to these inherent challenges in *Omics* data standardization, initiatives like the MF-PCBA data set^58^ actively promote data standardization and accessibility within the HTS domain, offering a valuable resource for developing and benchmarking AI methodologies for drug discovery; *Molecular Similarity* prioritizes drug design and lead-identification by quickly screening compound libraries, but it is limited to the chemical space from available information in databases and might miss compounds with more novel chemical properties and parameters for higher activity. Finally, *Network Pharmacology* tools generate an integration strategy and large perspective based analysis of disease targets that requires more standardized data integration parameters. They highlight systemic interactions and molecular/pathway relationships that guide novel biological understanding, yet they need strong models to test for those specific network interaction parameters to establish new hypotheses beyond those used by common and limited single drug–target approaches that are still heavily tested for novel compound development. Implementation of better methodologies for data transparency and bias control is a main path for future improvements across the board.

### 4.2 Theme 2: AI Methodologies for Lead Discovery, Optimization, and Hit Identification

AI models provide several methodologies for improving all drug-candidate related parameters and increasing efficacy with targeted approaches, which include: enhancing high-throughput screenings, generating structure-based molecule design approaches for better molecular interactions and properties, and repurposing available drug data by exploring new parameters. AI tools based on data models and algorithm designs provide more rational ways to approach all phases involved in complex parameters assessments for selecting high value pharmaceutical/medical entities in an efficient manner.

#### 4.2.1 AI Methodologies and Algorithmic Designs

AI-enhanced High-Throughput Screening (HTS) increasingly integrates neural networks, often employing CNN or RNN-based layers for rapid data extraction and analysis from high-content imaging assays. Furthermore, contemporary AI-driven HTS methodologies are leveraging transfer learning to effectively incorporate the inherent multifidelity of HTS data. Traditional HTS funnels generate data across tiers, from large-scale, lower-fidelity primary screens to smaller, high-fidelity confirmatory assays. Recent research, as exemplified by Buterez et al.,^58^ underscores the efficacy of transfer learning, particularly with Graph Neural Networks (GNNs), in enhancing molecular property prediction through the strategic integration of these multifidelity HTS data modalities. This approach acknowledges that primary screening data, while noisier, encompasses a vast chemical space, offering a valuable, inexpensive proxy to guide predictions on sparse, high-fidelity confirmatory screen data. Data sets like MF-PCBA,^59^ introduced by Buterez et al. to benchmark such methods, provide a publicly available collection of standardized multifidelity HTS data, facilitating the development and evaluation of AI/ML models that effectively utilize multitiered experimental information.

Virtual screening utilizes diverse AI algorithms, including deep learning models and GNNs, to prioritize candidate molecules based on predicted properties. MSGNN-DTA, for example, combines atom-, motif-, and protein-level graphs with gated skip-connections for robust binding affinity prediction, achieving a low RMSE of 1.237 on the KIBA benchmark and demonstrating practical utility in virtual screening acceleration through an FDA-approved drug case study.^59^

Structure-Based Drug Design (SBDD) with AI is increasingly leveraging Transformer architectures alongside GNNs, particularly for protein sequence analysis and binding site compatibility prediction. The LEP-AD framework, combining ESM-2 protein embeddings (derived from Transformer models pretrained on UniRef50) with GCNs, demonstrates state-of-the-art binding affinity prediction, achieving a 15% accuracy improvement over AlphaFold2-integrated models, highlighting the power of sequence-based embeddings.^60^ Furthermore, innovative approaches like DIFFDOCK,^61^ introduced by Corso et al., are revolutionizing SBDD. DiffDock reframes molecular docking as a generative modeling task employing diffusion models, shifting from regression-based pose prediction to learning the distribution of plausible ligand poses. This paradigm shift enables more nuanced representation of inherent uncertainty in molecular docking and the capture of multiple, distinct binding modes, surpassing the limitations of single-pose regression methods. Molecular docking, a cornerstone of SBDD and virtual screening, has thus witnessed transformative AI-driven advancements beyond traditional scoring functions and search algorithms.

AI-driven drug repurposing utilizes various machine learning methodologies, often employing supervised learning models like Random Forest or Support Vector Machines (SVMs) trained on drug activity data. More advanced approaches, like meta-learning frameworks such as Meta-GAT, are being explored to address data scarcity in repurposing by leveraging transfer learning methodologies across diverse drug and disease contexts, aiming to improve prediction accuracy, especially in low-data regimes.^62^ Large Language Models (LLMs) are also emerging as promising tools for drug repurposing. Frameworks like DrugReAlign, developed by Wei et al.,^56^ demonstrate that LLMs, when combined with multisource prompting, can effectively utilize vast knowledge bases to identify potential drug repurposing opportunities and mitigate challenges such as ’hallucinations’ through incorporating multisource information and spatial interaction data.

#### 4.2.2 Results from Published Studies

Studies show how AI enhances virtual HTS performance with speed and cost reductions while providing better evaluation for compounds by implementing specific data mining to allow selection of those that traditional screening methods were not fully optimized to discover; furthermore, AI leads to finding unique structures/compounds that were not previously evaluated or even tested using prior methods, while also showing improvement in potency and binding for target specificity in all model design implementation during new chemical and therapeutic compound and method development; AI-driven drug repurposing offers solutions that are fast/easily implemented when compared to more traditional drug discovery and have a larger applicability for high demand, unexplored therapeutic pathways.

#### 4.2.3 Model Limitations and Future Avenues

Current methods that are highly dependent on HTS data sets have implementation problems when there are not proper controls for all variables for the system to accurately mimic *in vivo* parameters, and they also present limitations for understanding protein interactions (since they are based mostly on screening with low biological system complexity or when using oversimplified testing conditions). Implementations that focus on structural based drug design have parameters limited by what is known about protein topology with bias relating to publicly known protein conformations (rigid models or limited dynamics representations) with difficulties that translate into the selection of limited or predefined chemical structures. Implementation bias comes from relying on data already reported, while overlooking areas outside explored/reported experimental data ranges that also have influence on how new drugs/scaffolds are developed (and therefore must be tested using new data implementations or parameters). Drug repurposing studies are also affected by the quality of data implementation, and the capacity to generate results for a specific target is affected by limitations of validation data sets for both previous or future use case implementations while also neglecting parameters that control clinical responses, or patient demographics (due to poor data set evaluations/implementation and use of inadequate methodology to validate new drug applications in repurposing strategies). It is necessary, therefore, to move from the current focus on “structural analysis and virtual assays” into models with multiple validation parameters including real *in vivo* models and clinical end point data parameters to make translation and implementation parameters reliable for drug design development and pharmaceutical innovations.

### 4.3 Theme 3: AI in ADMET, Toxicology, and Clinical Development

AI has become also very influential in evaluating parameters on how new drug candidates can show more safety, efficacy, better toxicity, and pharmacological responses within biological settings while also predicting clinical implementation in humanized models. The power of new data-driven AI models in ADMET/toxicity predictions allows not only streamlining the processes of preclinical trials, but also offering key data that are related to toxicity prediction, and parameters related to drug behaviors using multifactorial biological analysis to reduce costs/time while improving the success of pharmaceutical translation into clinical implementation, allowing safer testing parameters and more reliable model implementations. AI is also used during clinical phases to select better patients (or disease subtypes that benefit from that molecule) with reduced bias and less testing periods for achieving clinical goals that can benefit the whole clinical study so it can be more precise and robust based on the new AI tools and methodological frameworks.

#### 4.3.1 AI in Predictive Toxicology and Pharmacokinetics

AI excels at predictive toxicology and pharmacokinetics by leveraging diverse data and sophisticated architectures. For solubility and toxicity prediction, Transformer-based models, like those utilizing ChemBERTa and ProtBert embeddings, analyze molecular features to optimize drug properties, reducing preclinical attrition.^27,60^ ChemBERTa & ProtBert achieved an AUC of 0.973 on DTI prediction and demonstrated a 2–4% improvement in ROC-AUC over fingerprint-based methods for toxicity prediction, showcasing the value of transfer learning. For metabolic stability prediction, ProtBert embeddings have been successfully used to predict drug–enzyme interactions (e.g., CYP450), guiding structural modifications to avoid off-target effects.

In physiologically based pharmacokinetic (PBPK) modeling, GNNs enhance predictions by modeling tissue-specific partitioning coefficients. A recent GCN-based PBPK approach reduced simulation errors by 30% compared to traditional QSAR models by explicitly modeling blood-tissue barrier interactions.^63,64^

#### 4.3.2 AI in Clinical Trial Design and Development

AI is transforming clinical trial design and development, particularly through patient stratification and dose optimization. For patient stratification, AI tools like Trial Pathfinder analyze electronic health records (EHR) to optimize trial inclusion criteria. Trial Pathfinder, for instance, can simulate virtual trials across demographic subgroups, identifying enrollment strategies that reduce required sample sizes by 25–40% while maintaining statistical power. In oncology trials, has led to 35% faster enrollment and improved survival outcomes through machine learning-driven patient-trial matching.^62^

For dose optimization, GNNs are utilized to model pharmacokinetic parameters. GNN-based models can predict parameters like drug half-life, enabling optimized dosing regimens, although ongoing clinical validation is essential to fully realize this potential.^65^

To provide a clearer picture of the diverse AI methodologies and their demonstrated capabilities across various stages of drug development, Table 2 summarizes key examples of model architectures discussed in this review. This table highlights specific AI techniques, such as GNNs and Transformers, along with representative model names, typical parameter settings used in their implementations, and concrete examples of their application within drug discovery. Notably, it also includes performance metrics, like AUC and RMSE, where available from the referenced literature, offering a quantitative perspective on the efficacy of these AI approaches. When considering AI’s role in clinical trial design and development, as discussed in this section, and more broadly across ADMET, toxicology, and preclinical stages (Theme 3), Table 2 serves as a valuable reference, illustrating the tangible progress and varied architectures being employed to enhance drug discovery and development pipelines.

**Table 2:** Examples of AI Model Architectures and Performance in Drug Discovery

	Graph Neural Networks (GNNs)	Transformer Architectures	Meta-Learning
Example Architectures/Models	GCNs, GATs, RWGNN, DTI-HETA, MSGNN-DTA	BERT, Mol-BERT, LEP-AD, Transformer-BERT	Meta-GAT
Typical Parameter Settings (Examples)	Layers (multiple), Attention Heads (in GATs), Embedding Dimensions (e.g., 768)	Layers (e.g., 12), Attention Heads (e.g., 8–12), Embedding Dimensions (e.g., 768), Pretraining Data (e.g., SMILES)	Bilevel Optimization, Pretraining Tasks (e.g., molecular property prediction)
Example Application in Drug Discovery	Target Identification, DTI Prediction, Binding Affinity Prediction, Dose Optimization	Target Identification, Molecular Property Prediction, Toxicity Prediction, Binding Affinity Prediction	DTI Prediction in Low-Data Scenarios
Performance Metric (Example)	AUC (e.g., 0.957 for RWGNN), RMSE (e.g., 1.237 for MSGNN-DTA)	AUC (e.g., 0.973 for ChemBERTa), ROC-AUC improvements (e.g., 2–4%)	ROC-AUC (above 0.85)
Example Data Sets	DOCKSTRING, ExCAPE, BindingDB	QMugs, ChEMBL, ZINC	LIT-PCBA (for unbiased benchmarks)

#### 4.3.3 Advantages, Limitations, and Opportunities

AI implementation (via ML/DL) speeds up safety evaluation protocols, reduces reliance on costly/time-consuming animal testing, while also generating more targeted predictions with real data validation during the drug development process (based on parameters previously mentioned above).^35^ Further, AI applications based on AI driven algorithms facilitate the selection and stratification of patients based on multiple bioclinical/genetic parameters to evaluate and highlight those patients with the greatest chance of positive response, with overall more efficient use of financial resources with shorter implementation protocols in studies to generate more quality results by utilizing large, diverse data sets from real-world clinical observations with a higher degree of clinical implementation relevance and translatable parameters. Implementations are highly biased with limited models that do not access real *in vivo* data (with patients) and/or data models with very specific parameters relating to a single evaluation type that can lead to “oversimplification” in data models (or also over extrapolation or over reliance on model data output without testing all data set outputs and parameters, which may lead to dangerous clinical/pharmacological evaluations that miss key side effect information). Interpretability issues, lack of transparency or access to source codes with all parameters being open, create barriers to full implementation with bias issues, creating bottlenecks in wider utilization of and access to new methods. Future implementations require moving beyond animal models/highly simplified evaluations and starting to assess all biological/chemical parameters by having real patient output and implementation strategies using multi-parametric approaches, that are accessible to everyone for higher ethical implementation in clinical decisions during AI-based methods for drug implementations.

### 4.4 Theme 4: Challenges, Limitations, and Controversies

Despite the undeniable benefits and expanding possibilities of AI/ML across various facets of drug development and therapeutic applications, it is crucial to acknowledge the inherent methodological and technical biases that can currently limit their broader, real-world applications. Common concerns broadly fall into areas of data collection, method design, implementation workflow, and the crucial translation of AI insights into tangible clinical and biological results.

- **Data Quality and Accessibility: A Persistent Bottleneck in Real-World Application**. The high dependence of AI/ML model outputs on “pre-existing data” introduces fundamental biases that significantly impact data interpretations. In practice, access to high-quality, meticulously annotated, and comprehensively diverse data sets remains a major hurdle, especially for research groups operating with limited resources. This scarcity of robust data creates inequalities in access and skews model development and validation, potentially limiting the generalizability and reliability of AI-driven predictions. Furthermore, the inherent biases embedded within many publicly available data sets, often reflecting the demographics of studied populations or experimental biases in published literature, become amplified through AI/ML models. This can inadvertently lead to models that are less broadly applicable or, concerningly, perpetuate existing health disparities, reinforcing inequities in drug access and efficacy for underrepresented populations. Data sets often overemphasize certain trends at the cost of neglecting less frequent but potentially critical results, pathways, or side-effects, further skewing model outputs. While data sets with stringent validation protocols, transparency, and reproducibility are ideal, their relative scarcity poses a significant challenge to the widespread and equitable implementation of AI in drug discovery. As highlighted by Wei et al. in their DrugReAlign framework,^56^ biases arising from reliance on single or limited data sources are a key limitation that multisource prompting techniques attempt to address.
- **Addressing these data limitations is a focus of current research, resulting in the development of novel benchmark data sets designed to mitigate specific challenges**. For example, LIT-PCBA^66^ was specifically created to address the issue of hidden molecular biases in existing data sets, providing an ’unbiased’ resource for machine learning and virtual screening algorithm development. Similarly, the MF-PCBA^59^ data set tackles the challenge of data sparsity and noise inherent in High-Throughput Screening (HTS) data by incorporating *multifidelity* measurements (both primary and confirmatory screening results), offering a more comprehensive representation of experimental data. The DOCKSTRING^23^ data set directly tackles the practical challenge of benchmark accessibility and standardization in docking-based virtual screening by providing a user-friendly package, a large data set of docking scores and poses, and well-defined benchmark tasks. Furthermore, data sets like QMugs^67^ are emerging to address limitations in the scope and chemical space coverage of existing quantum chemistry data sets by providing a large collection of quantum mechanical properties for drug-like molecules, expanding the applicability of AI models in areas like molecular property prediction and *de novo* design. These data sets represent a shift toward creating more robust, representative, and accessible resources to drive further progress in AI-driven drug discovery, although challenges related to truly mirroring complex biological systems *in silico* remain.
- **Model Implementation Biases and the Challenge of the “Black Box” in Practical Workflows**. Inherent biases in model implementation, stemming from specific research parameters and algorithmic choices, significantly impede the practical application of AI. The “black box” nature of complex Deep Learning (DL) models particularly hinders real-world drug discovery workflows by creating barriers to effective troubleshooting and rigorous validation. When models fail or yield unexpected results, their lack of interpretability makes diagnosing the root cause and extracting actionable insights for iterative design exceedingly difficult, slowing optimization cycles. This opacity also undermines trust and broader adoption by medicinal chemists and pharmacologists, who require clear rationales behind AI predictions to confidently integrate these tools into their decision-making processes. As Zhou et al.^68^ emphasize, methods that overlook high-order neighborhood information in complex biological networks can limit prediction accuracy, highlighting the need for models that capture intricate topological features, beyond simple, direct interactions. To mitigate some of these interpretability and data representation challenges associated with standard Graph Neural Network approaches, Zhou et al. recently introduced JDASA-MRD, a model that integrates deep autoencoders with subgraph augmentation. JDASA-MRD aims to enhance representation learning and improve prediction accuracy, particularly in complex biological systems, by explicitly incorporating multihop neighborhood information and addressing issues like oversmoothing common in deep GNNs.
- **Financial Implications, Ethical Considerations, and Accessibility Barriers in Broad Implementation**. The financial implications of AI in drug discovery extend far beyond the initial costs of technology acquisition and implementation. The inequality in results accessibility, stemming from the complex methods, specialized teams, and high-performance computing infrastructure often required for effective AI utilization, can create a ’digital divide’ within the drug discovery landscape. This divide may disproportionately benefit well-funded institutions and large pharmaceutical companies with readily available AI expertise, potentially widening the gap between resource-rich industrial applications and academic research, and ultimately impacting global access to newly developed, affordable medicines. The trend toward proprietary AI methods and data sets further exacerbates these disparities, as valuable data and algorithms become increasingly siloed and inaccessible to the broader scientific community. Furthermore, ethical considerations surrounding data ownership, algorithmic bias, and the potential for misuse of AI in drug development necessitate proactive and ongoing interdisciplinary discussions. The establishment of robust ethical guidelines and adaptive regulatory frameworks is crucial to ensure responsible development and equitable access to AI-driven drug discovery technologies, preventing the exacerbation of existing health inequities. As Wei et al.’s EADTN framework^69^ implicitly acknowledges this by seeking more efficient and accessible models, addressing the computational and expertise barriers is critical for democratizing AI’s benefits in drug discovery.

#### 4.4.1 Challenges in Practical Implementation

Translating the promise of AI-driven drug discovery from research laboratories to routine practical application in the pharmaceutical industry presents a distinct set of challenges. While AI/ML models demonstrate impressive performance in controlled research settings, several hurdles remain for wider, seamless integration into established drug development workflows:

- **Integration with Existing Infrastructure and Workflows**: The seamless integration of AI/ML tools into existing pharmaceutical R&D infrastructure and established workflows remains a complex undertaking. Many pharmaceutical companies face the challenge of retrofitting AI solutions into legacy systems and adapting established experimental and data management processes to effectively leverage AI insights. This requires significant investment in infrastructure upgrades, data harmonization efforts, and the development of user-friendly interfaces that can be readily adopted by non-AI specialist scientists.
- **Validation and Regulatory Acceptance**: While scientific validation is a cornerstone of AI research, gaining regulatory acceptance for AI-driven drug discovery outputs presents a novel and evolving challenge. Regulatory agencies like the FDA and EMA are actively working to establish clear guidelines and validation parameters for AI/ML models used in drug development. However, standardized validation metrics, interpretability requirements for regulatory submissions, and clear pathways for demonstrating the reliability and robustness of AI-driven evidence are still under development. Bridging the gap between academic validation and the rigorous evidentiary standards required for regulatory approval is crucial for the widespread adoption of AI in practical drug development.
- **Skill Gap and Training**: The successful implementation of AI in drug discovery necessitates a workforce equipped with interdisciplinary skills, bridging the gap between traditional pharmaceutical sciences and AI/data science expertise. A significant skills gap exists in the pharmaceutical industry regarding the development, deployment, and interpretation of AI/ML models. Addressing this requires substantial investment in training and education programs for both AI specialists to understand pharmaceutical domain knowledge and for traditional pharmaceutical scientists to become proficient in utilizing and interpreting AI-driven tools and insights. Fostering effective interdisciplinary collaboration and communication is paramount for successfully integrating AI into routine drug discovery practice.

By proactively addressing these multifaceted challenges and focusing on open methodology principles, data transparency, and ethical considerations, the pharmaceutical field can responsibly and effectively unlock the full transformative potential of AI, paving the way for a new era of safer, more effective, and accessible medicines for all.

### 4.5 Compare and Contrast: AI Methodologies in Drug Discovery

AI-based approaches demonstrably accelerate drug development and reduce costs but necessitate strong ethical values and robust frameworks for transparency and bias control for proper implementation.

- **Target Identification**: GNN, text mining, and multiomics implementations each contribute uniquely to organizing existing knowledge. NLP excels at streamlining complex interconnections within textual data, linking knowledge parameters and pathways. Multiomics designs characterize novel drug mechanisms using biological/chemical parameters and pinpoint specific proteins for targeted drug design. Network topology analysis identifies crucial regulatory nodes and potential therapeutic targets. Model selection in target identification heavily depends on the specific data landscape and the chosen framework for integration. Deep neural network methods, including GNNs and Transformers, generally demonstrate strong performance, but are not without limitations. Specifically, they have data bias-related limitations that can influence final output selections and analyses and necessitate careful re-evaluation based on the chosen methodological approach.
- **Lead Discovery, Optimization, and Hit Identification**: AI’s capacity for high-throughput, low-cost approaches significantly accelerates lead discovery, optimization, and hit identification, while also enhancing the accuracy and predictiveness of chemical designs. However, current AI limitations often stem from an over-reliance on known structural motifs and a neglect of data from less explored chemical spaces, coupled with limited data set validity, particularly when based solely on *in vitro* assays. Robust “*denovo*” design methodologies leveraging diverse data sets and integrated AI/ML techniques are crucial to expand AI’s applicability and foster discovery beyond existing knowledge biases. DL models, especially when coupled with GNNs, are powerful predictive tools in this domain, and classical ML methods provide valuable frameworks for methodology design, initial data implementation, and parameter evaluation, with potential for refinement to improve model outputs and mitigate inherent biases. Recent advancements, exemplified by DiffDock, showcase the transformative potential of generative AI models in revolutionizing core techniques like molecular docking within lead discovery workflows.
- **ADMET, Toxicology, and Clinical Development**: AI-based tools, primarily data modeling tools, are increasingly valuable in ADMET, toxicology, and clinical development, though they are still evolving to handle the complexity of data beyond preclinical *in vitro* studies. AI applications in ADME parameter prediction and toxicity evaluation often necessitate further *in vivo* validation to address model limitations arising from the lack of comprehensive human/patient data and inherent biases.

#### 4.5.1 General Comparison and Performance Nuances

All AI/ML methods discussed perform well in specific aspects of drug discovery, offering improvements in speed, selectivity, specificity, and therapeutic potential. AI also enables more data-driven approaches in research, shifting away from purely expertise-based hypothesis generation in clinical study design and other areas. However, it is crucial to acknowledge that their superior complexity does not *always* translate to drastically better performance compared to classical Machine Learning methods like Random Forests and Support Vector Machines, especially in certain chemical modeling tasks. This nuanced perspective is supported by studies like Aleksić et al.^70^ who, in their ADMET predictability study, observed that simpler algorithms can sometimes achieve comparable or even superior performance to more complex deep learning models on certain ADMET end points. Similarly, Grötschla et al.^24^ in their benchmarking of positional encodings for GNNs and Transformers, highlight that, while modern architectures offer significant advancements, careful hyperparameter tuning and appropriate feature engineering for simpler models can sometimes yield surprisingly competitive results.

This suggests that, while GNNs and Transformers excel at capturing complex, nonlinear relationships in molecular data, the benefit of this complexity must be carefully weighed against factors such as data set size, data quality, and the specific task. In comparing GNNs and Transformers, recent work like Edge-Set Attention (ESA) suggests that purely attention-based, edge-centric approaches can potentially achieve state-of-the-art performance with potentially greater simplicity and scalability compared to encoding-heavy graph transformers, while also outperforming strong GNN baselines on many tasks.^14^ For certain drug discovery tasks, especially those with limited data sets or where interpretability is paramount, simpler, more transparent models may offer a pragmatic and effective alternative, while for tasks demanding high accuracy and the ability to learn from large, complex data sets, modern architectures like GNNs and Transformers remain at the forefront.

However, many methods, both classical and modern AI, still require substantial human expertise for complex scientific analyses, implementation protocol design for future data, and nuanced interpretation of results for therapeutic applications. AI has undeniably enhanced speed and selectivity across drug discovery, offering methodologies for improved target specificity and therapeutic effect potential, while also reducing resource expenditure and adapting research paradigms toward data-driven approaches rather than solely expertise-based hypotheses in clinical study design. AI applications, however, often lack robust evaluation of drug candidate behavior in diverse patient populations, sometimes focusing disproportionately on high-throughput screenings rather than addressing high data variability in individual patient responses. This variability, stemming from diverse social, biological, and clinical parameters, can limit the generalizability of models and their applicability across all patient subpopulations. Therefore, integrating clinical/translational validation as an essential parameter in future AI-driven drug discovery studies is paramount. Moving beyond solely *in silico* evaluations as core implementation data and emphasizing real-world clinical parameters are key to further refining method evaluation and design in current AI drug pipelines.

## 5 Conclusion

This review has explored the transformative potential of Artificial Intelligence (AI) and Machine Learning (ML) in drug discovery, while also acknowledging the critical challenges, complexities, and ethical issues that need to be solved to promote safe implementation with a focus on robust and valid AI models that must become ethical, accessible, valid, and useful for all patient populations in therapeutic pharmaceutical design pipelines. AI/ML integration streamlined the drug development pathway, speeding up and improving lead molecule/target identification by accelerating HTS methods, using innovative molecule designs, and also showing potential for safer drugs by using bio/data and biomodel implementations for evaluation, which allow repurposing of existing drugs based on known data with a new understanding of disease mechanisms by implementing parameters that did not exist during their first implementation. AI model/implementation parameters have indeed enhanced and optimized efficiency throughout the process while opening new scientific directions, providing also validation parameters that promote clinical/biological results while improving drug safety and decreasing development time or resources.

### 5.1 Summary of Key Points

Key insights obtained through this analysis include:

- **Transformative Potential of AI/ML**: AI/ML demonstrates a strong capacity for tackling persistent limitations in traditional drug discovery with AI methods designed for different objectives. Data validation with different tools and AI model designs also allows exploration of more parameters, as well as better methods selection with higher performance in lead compound selections in model implementation strategies for scientific research. The main challenge involves data bias that often favors only known data set results over the vast potential for drug design using previously unexplored pathways.
- **Diverse AI/ML Methodologies**: A growing number of AI methods are being implemented for various pharmaceutical goals in target evaluations, lead optimization, and other safety/efficacy tests. Those methods use different mathematical/algorithmic approaches, with methods and implementation designed according to the type of data sets or data goals for the specific stages of pharmaceutical research (with implementation ranging from CNNs and GNNs to Transformers and other methods for dimensionality reduction). New designs and adaptations of classical models must be encouraged since these methods have low computational barriers or design parameters.
- **Emphasis on Data and Validation**: Models that show translatable results are often related to better data set validation, also with proper use of benchmarks parameters. Implementation must include quality data evaluations from independent researchers with different viewpoints (and data set implementations). Those methods that have transparency, robust implementation with real *in vitro* testing data sets, and that also use validation results are better indicators of proper validation of AI methods to translate model and data into clinical implementation parameters. Ethical parameters (including clear statements on methodology implementation, code access, and open data set or robust data evaluations/accessibility) must be the core focus while selecting models that have a greater potential to influence how new drugs are made based on transparent methodologies that are understandable by diverse scientific communities in drug implementation.
- **Ethical and Regulatory Considerations**: Implementation must consider all parameters that prevent biases or limitations caused by oversimplifying model implementation or methodology implementation through ethical evaluations based on design parameters to promote transparency, accountability, and a strong foundation on robust data selection/implementation methodologies that can create methods for all pharmaceutical development processes that must also be compatible with diverse and variable patient populations/clinical conditions and not favor single-point parameter analysis. New regulatory frameworks are needed to improve methods for all areas of pharmaceutical development based on AI usage.
- **Future Directions**: The need to implement XAI methods/models is high, as well as the exploration and further development of multimodal data integration parameters and methods. Validations based on in vivo/clinical settings are critical to improve the accuracy of the design and model reliability/translatability (for methods). Clear methodologies are a key component of reliable scientific information that has better ethical translatable designs that create tools useful for diverse applications to all types of users across varied settings.

### 5.2 Identified Gaps

Key limitations identified across various reports reviewed highlighted the following needs:

- **Accessibility to Curated Data and Addressing Bias**: Proprietary or highly limited data sets create inequalities in access and biases in implementations and model outcomes due to the use of limited samples; lack of data transparency and diversity representation limit AI efficacy. Clear methodology for bias control, evaluation from different perspectives in the data set parameter, as well as source information (from data providers, study design, patient populations, etc.) is key for new methods. Implementation should prioritize data set sources that are readily available and open source or for those which methods for data set design or model evaluations can be openly checked and analyzed to test their translatable abilities with proper peer evaluation strategies. However, significant progress is being made in creating publicly available, high-quality data sets specifically designed to address these challenges. Resources such as LIT-PCBA directly target bias reduction in virtual screening benchmarks, MF-PCBA provides multifidelity HTS data to improve representation of experimental complexities, DOCKSTRING offers a standardized and accessible docking benchmark, and QMugs expands the chemical space and property coverage for quantum machine learning. Continued development and broader adoption of such meticulously curated and openly accessible data sets are crucial for advancing the field.
  ○ **Interpretability and Transparency of Implementation Methods**: Methods that implement “black-box” parameters present interpretation limits in how AI arrives at certain conclusions. More emphasis on “white box” implementations with models that have interpretable outputs using methods such as XAI (explainable AI) should be prioritized for broader implementation and method validation and are vital to achieve proper translation of model-driven outputs to new knowledge that promotes confidence in novel AI-driven drug pipelines in medical areas.
- ***In vivo* and clinical implementation**: Enhanced implementation parameters for complex, data-driven assessments—focusing on real-world application in preclinical studies and clinical trials to validate *in vitro* assays and model parameters—are needed to ensure performance outside computational simulations and to assess robustness and translatability through rigorous validation benchmarks. Moreover, model performance metrics must incorporate *in vivo* validation to capture not only binding affinities and potency in controlled assays, but also to predict real-life applicability and patient-specific responses within a multisystem biological context that includes human components. Finally, AI-driven data sets integrating preclinical validation results, patient-specific parameters, and therapeutic outcomes are vital for rigorously testing methods and developing more precisely targeted pharmaceutical protocols.

### 5.3 Future Research Directions

Given the aforementioned gaps, areas for research should include:

- **Data Standardization**: The creation of publicly available, open-source platforms with well-defined parameters and documentation should be encouraged and supported by governing and research institutions. Such platforms promote the sharing of high-quality, consistent data sets that adhere to standards for data validation, methodological transparency, and accessibility—including multilingual support to ensure equitable data access. Initiatives like LIT-PCBA, MF-PCBA, DOCKSTRING, and QMugs exemplify the critical impact of standardization in AI-driven drug discovery. Future efforts must continue to prioritize data quality and robustness, clear documentation, and open methodologies to accelerate progress across diverse research settings.
- **Advanced Docking Methodologies**: Continued exploration of generative modeling—particularly for molecular docking—is poised to drive the next wave of AI-driven lead discovery. Approaches like DiffDock, which reconceptualizes docking as a generative diffusion process, have demonstrated superior accuracy and an ability to capture intricate protein–ligand interaction features compared to traditional regression- or search-based methods. Future work should expand these generative docking frameworks, focusing on enhancements in precision, computational efficiency, and seamless integration with broader AI-driven drug-discovery pipelines.
- **Explainable AI (XAI)**: Continued research into explainable AI is essential for rendering model outputs transparent—showing how specific predictions are derived, identifying limitations before data deployment, and uncovering novel associations that require external or multisystem validation. Translating “black-box” outputs into interpretable components will enhance methodological understanding among scientific specialists and empower policymakers and other stakeholders with clearer insights into AI-driven discoveries.
- **Multimodal Data Integration**: Integrating multimodal inputs—including bioassay measurements, validated clinical end points, and diverse *in vivo* or animal model results—ensures that model outputs reflect complex biological and physiological contexts rather than simplified computational abstractions. Emphasizing patient-centered metrics (e.g., pharmacodynamic responses and toxicity profiles) across broad populations—including rare, underrepresented, and hard-to-reach subgroups—enhances the robustness, generalizability, and real-world applicability of AI-driven predictions.
- **Translational Data for Clinical Parameters**: Incorporating *in vivo* data—from cell-based assays and animal studies to real-time patient measurements—into AI model development is critical for clinical relevance. Models trained exclusively on *in vitro* or purely computational data often fail to predict patient-level outcomes; therefore, pipelines that integrate clinically relevant end points under rigorous safety and ethical protocols are essential to validate performance in real-world settings. Moreover, assessing scalability, ensuring compatibility with limited-infrastructure environments, and democratizing data access will facilitate equitable deployment of AI-guided drug discovery and support the creation of targeted therapies for diverse populations.

### 5.4 Closing Statement

Artificial intelligence and machine learning are now central to pharmaceutical innovation, demonstrably accelerating processes, reducing costs, and shortening timelines in drug development. This review underscores that realizing AI’s transformative potential hinges on stringent, ethically grounded validation methodologies. While AI tools promise better, safer, faster, and more accessible medicines by moving beyond theoretical parameters, achieving this requires clear standardization, regulations prioritizing scientific validity with interpretable outputs, and rigorous mitigation of model biases through ethical principles and data integrity. This review contributes to this crucial evolution by providing an in-depth comparative analysis of AI methodologies across the drug discovery pipeline, from target identification to clinical development, with a particular emphasis on practical challenges and future directions and on its critical evaluation of the integration of diverse data types and model architectures, offering a nuanced perspective on their strengths and limitations. Promoting interdisciplinary collaboration and transparency, we can ensure AI’s benefits are fully realized responsibly, creating safe, effective, and accessible medicines for a diverse global population, built upon the core foundations of ethical practice, robust validation, and technological accessibility.
